# Dietary Supplementation with Olive Mill Wastewater in Dairy Sheep: Evaluation of Cheese Characteristics and Presence of Bioactive Molecules

**DOI:** 10.3390/ani10111941

**Published:** 2020-10-22

**Authors:** Raffaella Branciari, Roberta Galarini, Dino Miraglia, David Ranucci, Andrea Valiani, Danilo Giusepponi, Maurizio Servili, Gabriele Acuti, Mariano Pauselli, Massimo Trabalza-Marinucci

**Affiliations:** 1 Department of Veterinary Medicine, University of Perugia, Via San Costanzo 4, 06126 Perugia, Italy; raffaella.branciari@unipg.it (R.B.); gabriele.acuti@unipg.it (G.A.); massimo.trabalza@unipg.it (M.T.-M.); 2 Istituto Zooprofilattico Sperimentale dell’Umbria e delle Marche “Togo Rosati”, Via G. Salvemini 1, 06126 Perugia, Italy; r.galarini@izsum.it (R.G.); a.valiani@izsum.it (A.V.); d.giusepponi@izsum.it (D.G.); 3 Department of Agricultural, Food and Environmental Sciences, University of Perugia, Borgo XX Giugno 74, 06121 Perugia, Italy; maurizio.servili@unipg.it (M.S.); mariano.pauselli@unipg.it (M.P.)

**Keywords:** polyphenols sulphate metabolites, spray-dried polyphenol compound, oxidative status, cheese, sheep

## Abstract

**Simple Summary:**

Using polyphenols from olive oil waste as feed supplements in animal diets can be a strategy to reduce adverse environmental effects of this by-product and to enhance the quality of products of animal origin. The aim of the study was to assess the effects of adding a polyphenolic concentrate from olive oil wastewater to a typical sheep diet on the nutraceutical and quality characteristics of cheese. The experiment was carried out on thirty-six Sarda ewes, divided into two homogenous groups fed a standard diet composed of hay and concentrate. In one of the two diets, the concentrate was supplemented (25 g/kg) with polyphenols obtained from olive mill wastewater using a special filtration system. Data showed that the polyphenol supplementation in the ewe’s diet resulted in the presence of tyrosol and hydroxytyrosol sulphate metabolites in milk and cheese. Furthermore, these compounds were able to provide a direct antioxidant effect on cheese with no modification in its chemical composition.

**Abstract:**

The aim of the study was to define the chemical characteristics, antioxidant capacity, oxidative status, sensory properties, and the presence of polyphenols in ovine cheese obtained after dietary administration of spray-dried olive mill wastewater (SDP). SDP is a waste from olive oil production rich in bioactive molecules obtained by further processing the olive mill wastewater through a spray-drying system. Thirty-six sheep were randomly assigned to two experimental groups that received a standard diet based on hay and concentrate. The concentrate fed to the SDP group was supplemented with SDP at a rate 25 g/kg (as fed). The trial lasted 9 weeks. Milk from the two treatment groups was separately collected and used for manufacturing cheese. Cheese quality parameters and proximate composition were not affected by the dietary treatment, whereas the antioxidant status and oxidative stability of cheese were positively affected. Polyphenol analyses in cheese were performed through liquid chromatography coupled to tandem mass spectrometry (LC-MS/MS). The concentration of hydroxytyrosol and tyrosol, and their sulphate metabolites, were higher in cheese from supplemented sheep. These findings suggest that polyphenol metabolites can play a major role in the beneficial effects observed in food produced from sheep fed SDP.

## 1. Introduction

The production of olive oil is widespread around the world but is particularly located in the Mediterranean region. According to the International Olive Oil Council (IOOC), 67% of the world’s olive oil production occurs in Europe, and Italy is one of the largest producers in the world [1]. Olive oil extraction generates adverse environmental effects with the production of a variety of by-products, depending on the process used; solid residues (olive cake) and liquid residues (olive mill wastewater) are produced with a traditional method through discontinuous press extraction and continuous three-phase extraction, while very wet residues represented by the olive wet cake are produced with the two-phase centrifugation process [2,3,4]. Olive cake is usually used to obtain biomass fuel, processed for soil conditioning and composting or included in ruminants’ diets. Olive mill wastewater and olive wet cake are environmental pollutants because of their high organic and phenolic content and have phytotoxicity activity [4].

New techniques for the treatment of these by-products have been developed to recover the highly valuable bioactive compounds found in olive oil waste [5]. The most representative bioactive molecules are phenolic compounds: (a) hydroxytyrosol (3,4-DHPEA) and tyrosol (*p*-HPEA) (class of phenolic alcohols); (b) dialdehydic form of decarboxymethyl elenolic acid linked to 3,4-DHPEA or *p*-HPEA (3,4-DHPEA-EDA or *p*-HPEA-EDA) (class of secoiridoids derivatives); (c) verbascoside (a derivative of hydroxycinnamic acid); (d) caffeic acid, *p*-coumaric acid, and vanillic acid (class of phenolic acids and derivatives); (e) lutein (class of flavones); and (f) (+)-acetoxypinoresinol and (+)-pinoresinol (class of lignans) secoiridoids, especially 3,4-DHPEA-EDA and verbascoside [6]. Because of their properties, these phenolic compounds can be used from a biological and pharmaceutical point of view in cosmetics and medicine and as nutraceutical products and antioxidants in foods [3]. Olive oil by-products can be used as feed supplements due to their capacity to reduce oxidative stress and improve antioxidant status, oxidative stability, shelf life, and quality characteristics in chicken [6,7,8] and rabbit [9] meat, beef [10,11], pork [12], and water buffalo [13] and cow [14] milk cheese. Their inclusion in animal diets could be a strategy to valorize olive oil waste and at the same time reduce environmental impact.

Our hypothesis was that supplementing the diet of lactating ewes with olive oil industry by-products might enrich milk and milk-derived products of bioactive compounds able to improve their nutraceutical and quality characteristics. Based on this hypothesis, the aim of the present work was to reveal the presence of phenolic compounds and their effects in cheeses derived from the milk of Sarda ewes fed with a concentrate enriched with spray-dried phenolics from olive mill waste-waters.

## 2. Materials and Methods

### 2.1. Animals and Diets

The study was performed at the Teaching Farm of the University of Perugia and was carried out in compliance with the European animal safety guidelines used for research purposes (EU Directive 2010/63/EU, 22 September 2010).

Thirty-six Sarda ewes were subjected to estrus synchronization and natural mating. The ewes were randomly divided into two homogeneous groups of 18 animals each at 60 d to the expected date of parturition, balanced by age, body condition score, and parity. The experimental treatments were represented by: (1) a basal control diet (C) composed of hay and concentrate; (2) a C diet where the concentrate was supplemented with olive mill wastewater (25 g/kg) treated with a filtration system consisting of progressive permeability membranes and dehydrated using a spray-drying system (SDP: spray-dried phenolics) (Table 1). The age of the animals ranged from 3 to 5 years, and parity ranged from 3 to 4. Average body condition score at the enrolment was 2.8 and 2.9 on a five-point system for C and SDP group, respectively. The study ended at approximately 110 d of lactation. During late pregnancy, the animals received 400 g concentrate/d, while during lactation, they were fed 800 g/d of concentrate in two equal portions during milking. The forage consisted of alfalfa hay (crude protein: 16.5 g/100 g; neutral detergent fiber (NDF): 38.8 g/100 g) administered ad libitum. Feed intake in the two dietary groups was similar. Average hay intake was 1.8 kg/day/head, and the amount of concentrate administered per day was ingested completely. Lambs were weaned at 40 d of age. The sheep were housed in a stable with access to an outdoor paddock for the entire length of the study.

### 2.2. Cheesemaking

At weeks 9 and 12 of lactation, milk from the two treatment groups obtained in two consecutive days was collected in two different refrigeration tanks and used for manufacturing cheese. Briefly, before milking, each experimental group was subdivided into 3 sub-groups (milking groups), which were milked separately. The Pecorino cheeses obtained from the three batches of milk derived from each dietary group of animals were manufactured separately using small-scale manufacturing facilities. The cheesemaking was conducted as reported by Pecorelli et al. [15] and a total of 18 cheeses (approximately 1.2 kg each) per dietary treatment (3 cheeses × 3 milking groups × 2 sampling periods) were obtained.

On average, the bulk milk pH was 6.64 and 6.68 for SDP and C, respectively, and contained 4.56% and 4.61% *w*/*w* lactose, 6.58% and 6.55% *w*/*w* fat, 5.32% and 5.28% *w*/*w* protein (MilkoScan 6000, Foss Electric, Hillerød, Denmark), and 552,000 and 616,000 somatic cells/mL (Fossomatic 5000, Foss Electric, Hillerød, Denmark), respectively.

### 2.3. Physicochemical Analysis of Feed and Cheese

All feeds were analyzed for dry matter (DM) [16], crude protein and crude fat [17], NDF, and acid detergent fiber (ADF) [18]. Sodium sulphite was used in the NDF procedure, and both the NDF and ADF are expressed inclusive of ash.

Physicochemical analyses of cheese were carried out on samples taken at 45 d of ripening. A puncture electrode probe connected to a portable pH meter (model MP120 Mettler Toledo Inc., Columbus, OH, USA) was used for the measurement of the cheese’s pH. Protein, fat, and ash content were determined according to AOAC methods (991.20, 995.19, 935.42) [16]; the Volhard method was used for determining the salt content (AOAC 2000 method 935.43) [16].

Feed and cheese fatty acids were extracted according to Branciari et al. [19], and the lipids were then esterified following the method described by Branciari et al. [20]. Fatty acid methyl esters were separated and quantified using a Perkin–Elmer AutoSystem-XL gas chromatograph equipped with a flame ionization detector (FID) and a split-splitless injector. Analyses were conducted with a CP-Select CB for FAME fused silica capillary column (100 m × 0.25 mm i.d., film thickness 0.39 µm, J&W, Agilent Technologies, Palo Alto, CA, USA). The injection volume was 1 µL. The carrier gas was high-purity helium with a flow rate of 1 mL/min. The injector and detector temperatures were kept at 290 °C. The column oven temperature was programmed at 120 °C increasing by 3.2 °C/min up to 170 °C and then increasing by 2.1 °C/min from 170 to 225 °C. Fatty acids were identified by comparison with standards as described by Branciari et al. [20].

### 2.4. Analysis of Polyphenols and Their Metabolites in Feed, Milk, and Cheese

Feed: Samples were prepared as described by Branciari et al. [6]. Briefly, 5 g of minced feed was extracted twice (2 × 25 mL) with a methanol/water 80/20 (*v*/*v*) mixture containing 20 mg/L of Butylated hydroxytoluene (BHT). Two aliquots of the reunited extracts were collected and diluted 50- and 500-fold, respectively, with a 0.1 M Na_2_EDTA/methanol 90/10 (*v*/*v*) mixture. After filtration, both aliquots were injected into a liquid chromatography tandem mass spectrometer (LC-MS/MS).

Milk: In a 50 mL falcon tube, 5 mL of milk was acidified at pH 4.6 with 1.5 M citric acid. Then, 10 mL of MeOH was added. After shaking and centrifugation, the supernatant was transferred and the extraction repeated with 10 mL of a methanol/water 80/20 (*v*/*v*) mixture containing 20 mg/L of BHT. The reunited extracts were evaporated to about 7 mL and loaded onto an SPE OASIS HLB cartridge (200 mg/6 mL, Waters, Milford, MA, USA) previously conditioned with 6 mL of methanol and 6 mL of 2 M acetic acid (pH 2). After washing (6 mL of water), the analytes were eluted with 6 mL of methanol. The eluate was defatted with 6 mL of hexane, evaporated, and resuspended in 1 mL of 0.1 M Na_2_EDTA/methanol 90/10 (*v*/*v*) solution. After filtration, the sample was injected into the LC-MS/MS.

Cheese: An amount of 1.0 g of cheese was extracted with 3 mL of a MeOH/H_2_O 80/20 (*v*/*v*) mixture containing 0.5% formic acid. After sonication and centrifugation, the extract was frozen at −80 °C for 30 min. The sample was then centrifuged at 0 °C and the supernatant transferred into a 15 mL falcon tube. The extraction process was exactly repeated with a further 3 mL including the freezing step. The reunited extracts were dried under a nitrogen stream and resuspended in 10 mL of 0.1 M Na_2_EDTA/methanol 90/10 (*v*/*v*) mixture prior to the LC injection.

### 2.5. LC-MS/MS Conditions

For tyrosol (T), hydroxytyrosol (HT), pinoresinol, and verbascoside, the LC-MS/MS conditions were set as described elsewhere [6]. For the determination of metabolites (tyrosol sulphate, T-S, hydroxytyrosol-3-sulphate, HT-3-S, and hydroxytyrosol-4-sulphate, HT-4-S), the same equipment was used, i.e., an LC-MS/MS system consisting of a Finnigan Surveyor LC pump, combined with a triple quadrupole TSQ Quantum Ultra triple quadrupole (Thermo Scientific, San Jose, CA, USA) with an electrospray ionization (ESI) interface operating in negative mode. Chromatographic separation was performed on a Gemini analytical column (100 mm × 2.0 mm, 3 μm, Phenomenex, Torrence, CA, USA) with water with 0.1% formic acid (A) and methanol (B) as mobile phases. The gradient started with 5% eluent B for 1 min and linearly increased to 35% B in 3 min, followed by a linear increase to 95% B in 13 min. After 6 min, the system decreased to 5% B in 1 min and was re-equilibrated for 6 min (run time: 30 min). The column temperature was 30 °C and the sample temperature was kept at 16 °C. Flow rate and injection volume were 0.25 mL/min and 10 μL, respectively. Nitrogen was used as sheath (30 arbitrary units) and auxiliary gas (20 arbitrary units). The collision gas was high-purity argon at 1.5 mTorr. The spray voltage was set at −2.5 kV and capillary temperature at 200 °C. The SRM transitions were *m*/*z* 217 >137 (T-S) and *m*/*z* 233 > 153 (HT-3-S, HT-4-S) [21].

### 2.6. Evaluation of Oxidative Status in Cheeses

The oxidative status of the cheese was evaluated measuring the secondary lipid oxidation products through the evaluation of thiobarbituric-acid-reactive substances (TBARS) using the method of Branciari et al. [22]. Briefly, 8 mL of phosphate buffer aqueous solution at pH 7 was mixed with 4 g of cheese in a 25 mL Sovirel tube, and the resulting mixture was homogenized using an Ultra-Turrax T 25 BASIC (Ika-Werke, Staufen, Germany). Two milliliters of a 30% (*v*/*v*) trichloroacetic acid aqueous solution were added, and the sample was homogenized followed by filtration with Whatman no. 1. Five milliliters of 0.02 M aqueous solution of thiobarbituric acid were added to 5 mL of the filtrate. The solution was put into capped tubes and heated for 40 min to 100 °C, then refrigerated. The absorbance of the supernatant was measured at 532 nm using an Ultrospec 2100 pro UV/visible spectrometer (Amersham Pharmacia Biotech, Little Chalfont, UK). Solutions containing 1,1,3,3-tetramethoxypropane (Sigma Aldrich, St. Louis, MO, USA) with a concentration range of 0.045 to 0.154 mg/mL (y = 2E + 07x + 0.0046; R² = 0.9999) corresponding to the range of 0.01 to 0.250 mg of malondialdehyde (MDA)/kg of cheese were used for the calibration curve for the quantitative determination of TBARS. The TBARS concentration was expressed as mg MDA/kg cheese. The determinations were carried out in triplicate.

### 2.7. Determination of Antioxidant Capacity of Feed and Cheese

The antioxidant capacity of feed and cheese was determined using DPPH and ABTS assays. Feed samples (1 g) were mixed with ethanol/0.1 M HCL 9.9/0.1 (*v*/*v*) solution at pH 4.0, while cheese samples (1 g) were mixed with 25 mL of MeOH/0.1 M HCL 9.9/0.1 (*v*/*v*) solution at pH 4.0. Each mixture (feed and cheese) separately was homogenized with an Ultra-Turrax homogenizer (Ultra-Turrax T25 Basic, IKA Labortechnik Janke & Kunkel GmbH, Stavfen, Germany) for 1 min and then vortexed for 2 min. The homogenates of feed and cheese were finally centrifuged at 6000 rpm at 4 °C for 20 min, and the supernatant of each was used for both DPPH and ABTS assays. The DPPH free radical scavenging activity of the extracts was measured using the method described by Branciari et al. [6]. The ABTS assay was done using the method of Blasi et al. [23]. The antioxidant capacity of each sample was expressed as milligrams of Trolox equivalents per gram (mg TE/g) of cheese. The calibration curve was built using (±)-6-hydroxy-2,5,7,8-tetramethylchromane-2-carboxylic acid standard (Trolox, 97%, Sigma) at different concentrations (from 0.1 to 0.5 mg/mL). All determinations were carried out in triplicate.

### 2.8. Colorimetric and Rheological Measurements in Cheese

For colorimetric and rheological determinations of cheeses, three samples (2 cm × 2 cm × 2 cm) from each cheese were obtained by cutting the inner part of the cheese with a sharp blade. The samples were immediately analyzed for surface color and texture profile measurements.

The surface color of the inner part of the cheeses was measured in triplicate using a Minolta Tristimulus Chromometer Minolta Chromameter CR400 (Minolta, Osaka, Japan—D65 light source calibrated against a standard white tile). The cooler measurements were performed after a 5 min bloom period at room temperature, and the results are expressed as lightness (L*), redness (a*), and yellowness (b*) (CIE Lab, 1986) [24].

The texture profile analysis (TPA) of compression testing [25] was carried out using a texture analyzer (TVT 6700, Perten Instruments, Milano, Italy) equipped with a 15 kg load cell and a 35 mm diameter cylinder flat-ended probe. Each sample was subjected to a double-cycle compression with the following parameters: test speed 2.0 mm/s; compression rate 30% of the sample high; trigger force 5 g; reaction speed 2.0 mm/s; rest period between the two compressions 5 s. Peak force (hardness expressed in N), cohesiveness, adhesiveness, gumminess, springiness (expressed in mm), chewiness (expressed in mJ), and resilience were obtained from the generated force–time curve, as reported in the Perten TexCal software manual (Perten, Milano, Italy, version 4.0.4 67).

### 2.9. Sensory Analysis

A group of panelists consisting of 30 assessors was recruited to evaluate if the samples of the two products (C and SDP) were perceptible different for organoleptic traits. For this purpose a “triangle test” following the ISO 4120:2004 [26] was performed, asking to choose the odd cheese tested in three separate sections (1 cheese for each treatment × 3 milking groups only for cheeses obtained from the 12 week of lactation milk). Panelists were recruited from the staff of the University of Perugia, previously educated in triangle evaluation techniques [27]. Prior to sensory evaluation, they took part in briefing sessions. They were asked to choose the odd cheese in a series of triangle tests.

### 2.10. Statistical Analysis

The effects of the diets (C and SDP) were tested with an ANOVA model using the GLM option in SAS (JMP 9; SAS Institute Inc., Cary, NC, USA, 2010)]. Factors included in the model were: sampling period (week and 9 and 12 of lactation), dietary treatment (C and SDP), and milking groups (3 per dietary treatment) within the dietary treatment. The interactions between main factors were not included in the model because were found to be not significant (*p* > 0.05). Data were reported as least square means ± standard error of the mean (SEM). Overall differences between treatment means tested according to the Tukey test (SAS, 2001) [28] and considered to be significant when *p* < 0.05. Tendencies were discussed when *p* > 0.05 but ≤0.10. Regarding the sensory data, to obtain a statistical significance at a level of 0.05 for both α-risk (probability of concluding that a perceptible difference exists when one does not) and β-risk (probability of concluding that no perceptible difference exists when one does) (ISO 4120:2004 [26]), 30 trained panelists were used.

## 3. Results

The effects of the lactation period and milking groups were not significant for all the parameters tested, and therefore, the results are reported only for dietary treatment groups.

### 3.1. Milk and Cheese Characteristics and Fatty Acid Profile

The pH and chemical composition of the cheeses is reported in Table 2. The pH values did not significantly differ between the two groups as well as the chemical composition. The mean values recorded are within the normal range for similar ewe-milk cheeses either for proximal analysis or NaCl content or pH [19,29].

The fatty acid composition of the samples is reported in Table 3. Total saturated fatty acid (SFA) content was higher (54.35% and 57.92% for C and SDP, respectively) than monounsaturated fatty acids (MUFA) (30.84% and 31.79% for C and SDP, respectively) and polyunsaturated fatty acids (PUFA) content (9.81% and 10.89% for C and SDP, respectively). In the case of SFA, 16:0 represented the most abundant fatty acid (24.76% and 24.89% for C and SDP, respectively), followed by 14:0 (9.04% and 8.93% for C and SDP, respectively) and 18:0 (9.07% and 8.81% for C and SDP, respectively). The most abundant individual MUFA was 18:1n-9 (20.75% and 20.96% for C and SDP, respectively), while PUFA were characterized by a high level of 18:2 n-6 cis (4.39% and 4.68% for C and SDP, respectively). Total SFA tended to be lower (*p* = 0.07) and total PUFA tended to be higher (*p* = 0.06) in the SDP milk, being C18:2n6c and C22:6n3 the two fatty acids with the highest increase (*p* = 0.052 and *p* = 0.017).

### 3.2. Phenolic Compounds in Feed, Milk and Cheese

The polyphenol content in feed and cheese is shown in Table 1 and Table 4. The four molecules determined in feed represent the main phenolic compounds present in olive mill by-products, showing high availability and bioactive activities, such as antioxidant properties.

**Figure 1:**
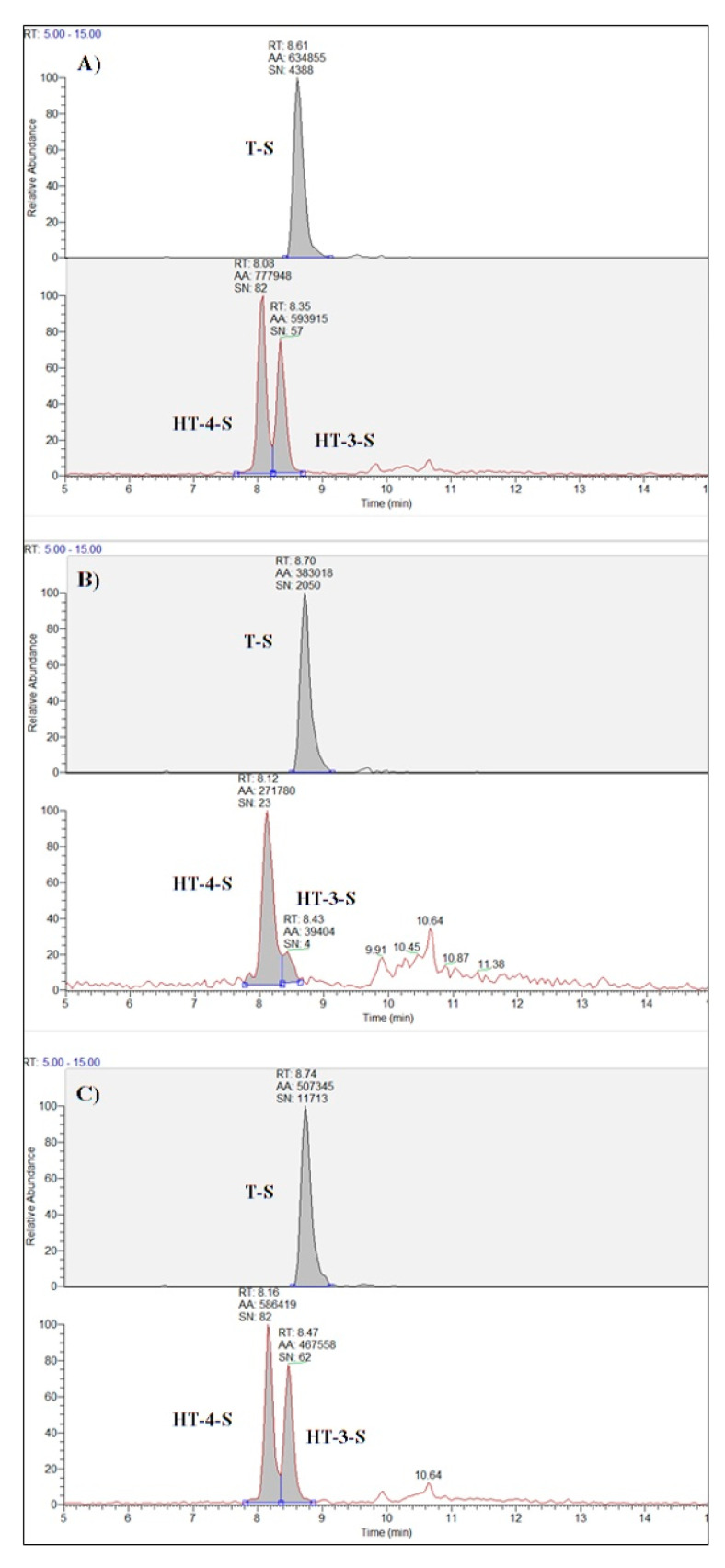
LC-MS/MS chromatograms of tyrosol (T-S) and hydroxytyrosol sulphates (HT-3-S and HT-4-S) in a matrix-matched standard at 100 µg/kg (**A**), in C Pecorino cheese collected at 45 days (**B**) and in the same sample spiked at 100 µg/kg (**C**).

The content of phenolic compounds in ewe’s milk used for cheese production was: tyrosol, hydroxytyrosol, and verbascoside 0.8, 0.3 and 0.20 µg/L, respectively, in the C group and 0.7, 2.1 and 0.3 µg/L, respectively, in the SDP group. Pinoresinol was not found, whereas sulphate metabolites were detected in milk at the following concentrations: tyrosol sulphate, hydroxytyrosol-3-sulphate, and hydroxytyrosol-4-sulphate 12, 7.4, and 11 µg/L, respectively, in the C group and 47, 230, and 291 µg/L, respectively, in the SDP group. The LC-MS/MS quantification of polyphenols in cheese (Table 4 and Figure 1) revealed significant differences between the two experimental groups only for sulphates metabolites. Higher levels of hydroxytyrosol-4-O-sulphate (HT-4-S), hydroxytyrosol-3-O-sulphate (HT-3-S), and tyrosol sulphate (T-S) were detected in SPD cheeses. Nonetheless small levels of such compounds were also registered in the control group. No differences were recorded for hydroxytyrosol (HT) and tyrosol (T) content between the two groups considered.

### 3.3. Oxidative Status and Antioxidant Stability in Cheese

The influence of dietary supplementation with SDP on oxidative status (TBARS) and on antioxidant stability (DPPH and ABTS) in cheese is presented in Table 5. The TBARS value of the C cheeses was higher than that of the SDP cheeses (*p* < 0.001) at the end of the ripening time. An increase in antioxidant activity was found in the SDP concentrate as well. The DPPH and ABTS results of the SDP cheeses were higher than those of the C cheeses (Table 5).

### 3.4. Colorimetric and Rheological Measurements

The SDP supplementation did not modify the color of cheese (Table 6), as no significant differences were detected for lightness (L* value), reddishness (a* value), and yellowishness (b* value). The results registered fall into the normal range for these kinds of cheeses made from sheep milk [19]. Moreover, the dietary treatment with SDP did not also affect the physical attributes, such as the texture or firmness of the final cheese product. Table 7 shows the texture profile of the cheeses. No significant differences were observed for any of the parameters studied.

### 3.5. Sensory Analysis

The results of the sensory “triangle test” reveal that only 10 judges, out of 30, could discriminate between the C and SDP cheeses. Therefore, the results were found not statistically different and no discrepancies in sensory characteristics between the two cheese groups were detected.

## 4. Discussion

The results of the chemical composition of the cheeses considered were not different, and these findings are in agreement with those of Abbeddou et al. [30], who found no variations in the composition of cheese following feeding of Awassi ewes with olive cake and olive leaves, and with Chiofalo et al. [14], who fed dairy Friesian cows with olive cake. Nonetheless, other authors registered variation in the proximal analyses of cheeses obtained from milk of animals feed with different sources of polyphenols. Taticchi et al. [13] found a higher percentage of fat in Mozzarella cheese obtained from lactating buffaloes fed dried stoned olive pomace, due to the amount of olive pomace in the concentrate that promoted this modification in the Mozzarella cheese. The dietary supplementation of Saanen goats with olive leaves, as reported by Innosa et al. [31], influenced the chemical–nutritional composition of Ricotta cheese, resulting in a lower level of fat compared to control samples. The lower amount of total fat registered in Ricotta cheese could be explained by an increase in total protein content of the whey [32].

Regarding the cheese fatty acid profile, a reduced ruminal biohydrogenation of PUFA was observed in vitro when phenol-rich forages were incubated in the presence of linseed oil [33]. Plant polyphenols are known to exert a negative effect on PUFA biohydrogenation by the rumen bacteria [34]. Pallara et al. [35] used stoned olive pomace in an in vitro fermentation system and observed a simultaneous decrease in *Butyrivibrio proteoclasticus* and an increase in vaccenic acid. The total phenolic content of the SDP concentrate was relatively low (552 mg/kg, considering the four major phenolic compounds analyzed), and no effect on ruminal biohydrogenation occurred in the present experiment. Abbedou et al. [30] reported changes in the fatty acid profile between control cheese and cheese obtained from milk of ewes fed olive cake (higher proportions of 18:1 cis and MUFA). According to the authors, the olive cake supplementation promoted 18:1 n-9 in milk by direct transfer from feed, since this fatty acid is not a biohydrogenation intermediate. Another study [13] reported a higher percentage of unsaturated fatty acids for a higher amount of oleic acid and a lower percentage of SFA in Mozzarella cheese obtained from lactating buffaloes fed dried stoned olive pomace. The authors explained that this could depend directly on the feed source and on the mammary gland desaturation activity. A study conducted by Chiofalo et al. [14] on cheese produced from supplementing dairy Friesian cows with olive cake showed a modification of fatty acids; indeed, the sample obtained with olive cake supplementation produced an increase in the PUFA and MUFA and a decrease in SFA in comparison with the control sample.

Although the presence of these metabolites (hydroxytyrosol-4-O-sulphate, hydroxytyrosol-3-O-sulphate and tyrosol sulphate) is well documented in biological fluids in human and animal tissues [36,37], to the best of our knowledge, this is the first study reporting their occurrence in milk and cheese. Our findings show that olive polyphenol supplementation determines the appearance in milk, and subsequently in cheese, of two hydroxytyrosol metabolites (isomers 3- and 4-sulphate), confirming that olive polyphenols are absorbed in the gastrointestinal tract [37]. It is worth noting that low concentrations of polyphenols were measured in the control samples as well. In this regard, previous studies have demonstrated that hydroxytyrosol and tyrosol are endogenously generated in small amounts as by-products of tyramine and dopamine metabolism. In particular, hydroxytyrosol is a product of dopamine oxidative metabolism known as DOPET (3,4-dihydroxyphenylethanol) [38,39,40]. Furthermore, the hydroxytyrosol and tyrosol bioavailability in biological fluid depends on an extensive metabolic pathway producing different metabolites such as glucuronide and sulphate conjugates. This study is in agreement with other research, which confirms that the concentrations of hydroxytyrosol and tyrosol in biological fluids are extremely low compared to those of their metabolites [36,41,42]. In particular, sulphates are the most abundant among the phase II metabolites [41,43], due to the action of enzymes, namely sulphotransferases, uridine 5′-diphosphoglucuronosyl transferases, catechol-O-methyltransferases, and acetyltransferases. The biological activity of hydroxytyrosol and tyrosol sulphates has recently been evaluated, showing that their antioxidant effect in intestinal cells (Caco-2) displays a protective efficiency comparable to that of the parent compounds [44].

Along with the modulation of oxidative status, the antioxidant activity of these compounds is also of great interest, since beneficial effects have been observed in several dietary intervention studies carried out on farms in different food-producing animal species. For example, Luciano et al. [45] and Branciari et al. [6,10] found extended oxidative stability of lamb, beef, and chicken meat, respectively, following olive cake dietary supplementation. By supplementing animals with extra virgin oil, Tufarelli et al. [8] determined an increased antioxidant defense system and reduced lipid peroxidation in chicken liver. Finally, Taticchi et al. [13] found an improvement of the dietetic and nutritional characteristics of Mozzarella cheese produced from the milk of buffaloes fed with dried stoned olive pomace. Regarding the differences registered in the TBARS values between the two cheese groups, these are consistent with the increase in antioxidant compounds in milk and therefore in cheeses. Terramoccia et al. [46] found that the milk of buffaloes fed a diet supplemented with dried stone olive pomace contained phenolic substances with powerful natural antioxidant activity, such as 3,4-DHPEA that positively contributed to lipid oxidation of the milk of treated animals by reducing the level of TBARS. Similar results were obtained by Taticchi et al. [13] in the governing liquid of Mozzarella cheese obtained from water buffaloes fed a diet supplemented with olive polyphenols. The authors explained these findings by the partial transfer of hydrophilic polyphenols, with high antioxidant activity, from cheese to liquid. Furthermore, an increase in antioxidant activity was found in the SDP concentrate and cheeses. The reduction in oxidative status and the improvement of antioxidant activity in products of animal origin obtained using diets supplemented with olive phenols is well documented in the literature [6,9,10,41]. These findings are related to the presence of powerful natural antioxidants occurring in olive oil by-products, acting either directly or indirectly against free radicals [47]. The hypothesis that polyphenols from olive mill wastewaters, supplemented in the diet of lactating animals in spray-dried form, can increase the presence of antioxidant compounds in cheese was confirmed.

Another important aspect for enriched food with functional attributes is the maintenance of typical sensory characteristics, for proper appreciation by the consumers [48]. In the present trial, no differences in the cheese color were detected between the two dietary groups. These results are in agreement with those of Innosa et al. [32] who supplemented the diet of goats with olive leaves and found no modification in the lightness (L*) of Ricotta cheese. A lack of effect of dietary supplementation with dried stoned olive pomace on color was also registered by Taticchi et al. [13] in Mozzarella cheese. The influence of polyphenols added to animal diet on the color of cheese is difficult to explain, because there are different factors able to influence L*, a*, and b* indices, such as the ingredients in the diet, especially the pigments contained in it, the fat percentage of the cheese, and the manufacturing process [32]. Even regarding the rheological traits, no differences were detected between C and SDP cheeses. Other authors have reported that phenolic compounds can affect cheese firmness by an indirect action. Abbedou et al. [31] fed ewes with olive cake and obtained less firm cheese, and a decrease in hardness with a lower cheese pH value, as suggested by Abd El Aziz et al. [49], affects the texture of curd directly by influencing the solubility of the caseins, because of the action of phenolic compounds in the cheese curd. Furthermore, a lack of effect on sensory traits was observed in the present trial, and this agrees with previous reports. No difference was registered by Taticchi et al. [13] in Mozzarella cheese obtained by supplementing the diet of lactating buffaloes with dried stoned olive pomace, or Roila et al. [50], who directly added a low dose (250 μg/mL) of olive oil polyphenols in cheese, since olive oil phenolic compounds bound to proteins are not easily perceived by the assessors. Chiofalo et al. [14] did not find significant differences between cheese made from the milk of cows supplemented with olive cake and control samples; however, consumers showed a greater preference for cheese obtained from the treated group.

## 5. Conclusions

The present study shows that olive aqueous waste can add a considerable amount of tyrosol, hydroxytyrosol, verbascoside, and pinoresinol to the diet of lactating ewes. For the first time, the presence of tyrosol and hydroxytyrosol sulphate metabolites in milk and cheese was demonstrated in an on-farm study. The addition of polyphenols to the ewes’ diet does not affect cheese composition; however, these compounds are able to provide a direct antioxidant effect, enhancing the nutraceutical value of cheese due to their well-known beneficial biological activity. Obtaining functional foods from olive mill wastewaters can be a valid option to give these polluting substances a high added value. Further research is needed in the light of decreasing the treatment costs of waste obtained from industrial oil extraction.

## Figures and Tables

**Table 1:** Ingredients (% as fed basis), chemical composition (g/100 g), fatty acids (g/100 g of total fat), and polyphenolic compounds (mg/kg).

Item	Concentrates	
	C	SDP
**Ingredients**		
Maize flour	24.58	23.58
Wheat bran	22.50	21.50
Wheat flour middlings	13.50	13.00
Soybean meal	13.50	13.50
Barley flour	9.00	9.00
Laminated linseed	9.00	9.00
Soybean oil	4.50	4.50
Calcium carbonate	1.44	1.44
Magnesium oxide	0.36	0.36
Sodium bicarbonate	0.90	0.90
Sodium chloride	0.45	0.45
Vitamin–mineral supplement ^a^	0.27	0.27
Spray-dried olive mill wastewater phenolics	0.00	2.50
**Chemical composition**		
Dry matter	88.40	88.53
Crude protein	15.93	15.48
Crude fat	10.21	10.49
Ash	6.30	6.97
NDF	21.56	21.57
ADF	5.74	5.66
ADL	1.55	1.42
Ca	1.30	1.45
P	0.81	0.77
**Fatty acids**		
C14:0	0.16	0.10
C16:0	14.57	13.74
C18:0	5.68	4.88
C18:1n9c	22.01	22.48
C18:2n6c	42.63	43.34
C18:3n3	13.79	14.53
C20:0	0.42	0.34
C20:1n9	0.34	0.29
C22:0	0.40	0.32
**Polyphenols**		
Hydroxytyrosol (HT)	0.07	272
Tyrosol (T)	0.32	37.1
Verbascoside	0.04	243
Pinoresinol	0.14	0.29

C, control; SDP, control with the addition of spray-dried phenolic compounds (25 g/kg) from olive mill wastewaters; NDF, neutral detergent fiber; ADF, acid detergent fiber; NFC, non-fiber carbohydrates; ADL, acid detergent lignin. ^a^ Vitamin–mineral supplement supplied per kg of diet: vitamin A, 18,000 I.U. (retinol); vitamin D3, 2100 I.U.; vitamin E, 21 mg (a-tocopheryl acetate); Fe, 29 mg; Co, 0.75 mg; Mn, 39 mg; Zn, 150 mg; Se, 0.06 mg.

**Table 2:** The effect of spray-dried polyphenol compound (SDP) supplementation on the chemical composition and pH of cheese after 45 days of ripening.

Cheese Composition	Dietary Treatment		SEM	*p*-Value
	C	SDP		
Moisture (g/100 g) *	42.58	42.28	0.18	0.574
Fat (g/100 g)	26.21	26.10	0.26	0.876
Protein (g/100 g)	26.73	27.10	0.31	0.672
Ash (g/100 g)	4.47	4.52	0.05	0.739
NaCl (g/100 g)	2.16	2.22	0.08	0.815
pH	5.28	5.31	0.01	0.325

C, control; SDP control with the addition of 25 g/kg of spray-dried phenolic compounds; SEM, standard error of the means; * g/100g of fresh weight.

**Table 3:** Effect of spray-dried polyphenol compound (SDP) supplementation on the fatty acid profile (g/100 g of total fatty acid methyl esters) of cheese after 45 days of ripening.

Fatty Acids	Dietary Treatment		SEM	*p*-Value
	C	SDP		
C4:0	4.15	3.92	0.04	0.079
C6:0	2.10	1.98	0.08	0.602
C8:0	1.59	1.49	0.05	0.475
C10:0	4.50	4.06	0.10	0.162
C12:0	2.66	2.46	0.06	0.284
C14:0	9.04	8.93	0.11	0.725
C15:0	0.94 ^b^	0.85 ^a^	0.01	0.002
C16:0	24.76	24.89	0.16	0.785
C17:0	0.56 ^b^	0.53 ^a^	0.00	0.001
C18:0	9.06	8.81	0.06	0.204
Total SFA	59.35	57.92	0.25	0.070
C14:1	0.17	0.17	0.00	0.567
C16:1	0.82 ^a^	0.86 ^b^	0.00	0.024
C18:1n9t	1.48	1.55	0.12	0.835
C18:1n7t	6.54	7.21	0.13	0.092
C18:1n9c	20.75	20.96	0.16	0.641
C18:1n7c	1.07	1.03	0.01	0.104
Total MUFA	30.84	31.79	0.16	0.180
C18:2n6t	1.45	1.35	0.03	0.358
C18:2n6c	4.39	4.68	0.04	0.052
C18:3n3	1.19	1.28	0.02	0.190
C18:2 9c11t	2.56	2.77	0.10	0.458
C20:4n6	0.07	0.07	0.00	0.647
C20:5n3	0.04	0.05	0.00	0.203
C22:5n3	0.07	0.08	0.00	0.286
C22:6n3	0.03 ^a^	0.04 ^b^	0.00	0.017
Total PUFA	9.81	10.29	0.08	0.060

C, control; SDP control with the addition of 25 g/kg of spray-dried phenolic compounds; SEM, standard error of the Means. Different superscript letters (a,b) in the same row define significant difference in the mean values (*p* < 0.05). SFA, saturated fatty acid; MUFA, monounsaturated fatty acids; PUFA, polyunsaturated fatty acids.

**Table 4:** Effect of spray-dried polyphenol compound (SDP) supplementation on the parent and metabolite compounds in Pecorino cheese after 45 days of ripening.

Phenolic Compounds	Dietary Treatment		SEM	*p*-Value
	C (µg/kg dw ^a^)	SDP (µg/kg dw)		
Hydroxytyrosol-4-O-sulphate (HT-4-S)	22 ^a^	773 ^b^	7.30	<0.001
Hydroxytyrosol-3-O-sulphate (HT-3-S)	14 ^a^	549 ^b^	9.19	<0.001
Tyrosol sulphate (T-S)	70 ^a^	176 ^b^	6.71	0.002
Hydroxytyrosol (HT)	29	33	4.12	0.772
Tyrosol (T)	613	808	46.42	0.232

dw, dry weight; C, control; SDP control with the addition of 25 g/kg of spray-dried phenolic compounds; SEM, standard error of the means. Different superscript letters (a,b) in the same row define significant difference in the mean values (*p* < 0.05).

**Table 5:** Effect of spray-dried polyphenol compound (SDP) supplementation on the antioxidant capacity and oxidative status of cheese after 45 days of ripening.

	Dietary Treatment		SEM	*p*-Value
	C	SDP		
DPPH (µmol TE/100 g)				
Concentrates	127.41 ^a^	245.73 ^b^	0.48	<0.001
Cheese	405.02 ^a^	430.50 ^b^	1.59	<0.001
ABTS (µmol TE/100 g)				
Concentrates	179.12 ^a^	533.47 ^b^	3.26	<0.001
Cheese	22.39 ^a^	49.64 ^b^	1.29	<0.001
TBARS (mg MDA/kg)				
Cheese	0.16 ^b^	0.10 ^a^	0.01	<0.001

C, control; SDP control with the addition of 25 g/kg of spray-dried phenolic compounds; SEM, standard error of the means. Different superscript letters (a,b) in the same row define significant difference in the mean values (*p* < 0.05). TBARS, thiobarbituric-acid-reactive substances.

**Table 6:** Results of color of cheeses after 45 days of ripening.

Item	Dietary Treatment		SEM	*p*-Value
	C	SDP		
L*	78.59	77.83	0.75	0.323
a*	−3.93	−3.80	0.08	0.235
b*	15.71	15.22	0.25	0.227

C, control; SDP control with the addition of 25 g/kg of spray-dried phenolic compounds; SEM, standard error of the means; L*, lightness; a* reddishness; b* yellowishness.

**Table 7:** Effect of spray-dried polyphenol compound (SDP) supplementation on the texture profile of cheeses after 45 days of ripening.

Attribute	C	SDP	SEM	*p*-Value
Hardness (g)	4118.23	3746.54	162.39	0.139
Springiness	0.76	0.76	0.00	0.299
Cohesiveness	0.70	0.67	0.01	0.089
Adhesiveness (J)	48.87	42.57	5.42	0.501
Gumminess (g)	2873.00	2524.85	125.70	0.084
Chewiness (g)	2179.46	1927.77	99.52	0.100

C, control; SDP control with the addition of 25 g/kg of spray-dried phenolic compounds; SEM, standard error of the means.
